# Nanomaterial-based drug delivery systems as promising carriers for patients with COVID-19

**DOI:** 10.1039/d1ra04835j

**Published:** 2021-08-02

**Authors:** M. Abd Elkodous, S. O. Olojede, Mahmoud Morsi, Gharieb S. El-Sayyad

**Affiliations:** Department of Electrical and Electronic Information Engineering, Toyohashi University of Technology Toyohashi Aichi 441-8580 Japan mohamed.hamada.abdlekodous.xi@tut.jp; Center for Nanotechnology (CNT), School of Engineering and Applied Sciences, Nile University Sheikh Zayed Giza 16453 Egypt; Nanotechnology Platforms, Discipline of Clinical Anatomy, Nelson Mandela School of Medicine, University of KwaZulu-Natal Durban South Africa; Faculty of Medicine, Menoufia University Menoufia Shebin El Kom Egypt; Drug Radiation Research Department, National Center for Radiation Research and Technology (NCRRT), Egyptian Atomic Energy Authority (EAEA) Cairo Egypt Gharieb.Elsayyad@eaea.org.eg; Chemical Engineering Department, Military Technical College (MTC) Egyptian Armed Forces Cairo Egypt

## Abstract

Once the World Health Organization (WHO) declared the COVID-19 outbreak to be pandemic, massive efforts have been launched by researchers around the globe to combat this emerging infectious disease. Here we review the most recent data on the novel SARS-CoV-2 pathogen. We analyzed its etiology, pathogenesis, diagnosis, prevention, and current medications. After that, we summarized the promising drug delivery application of nanomaterial-based systems. Their preparation routes, unique advantages over the traditional drug delivery routes and their toxicity though risk analysis were also covered. We also discussed in detail the mechanism of action for one example of drug-loaded nanomaterial drug delivery systems (Avigan-contained nano-emulsions). This review provides insights about employing nanomaterial-based drug delivery systems for the treatment of COVID-19 to increase the bioavailability of current drugs, reducing their toxicity, and to increase their efficiency.

## Abstract 2

We propose a consideration of Avigan-loaded nano-emulsions as a promising nano-based delivery system for the treatment of COVID-19.

## Graphical abstract

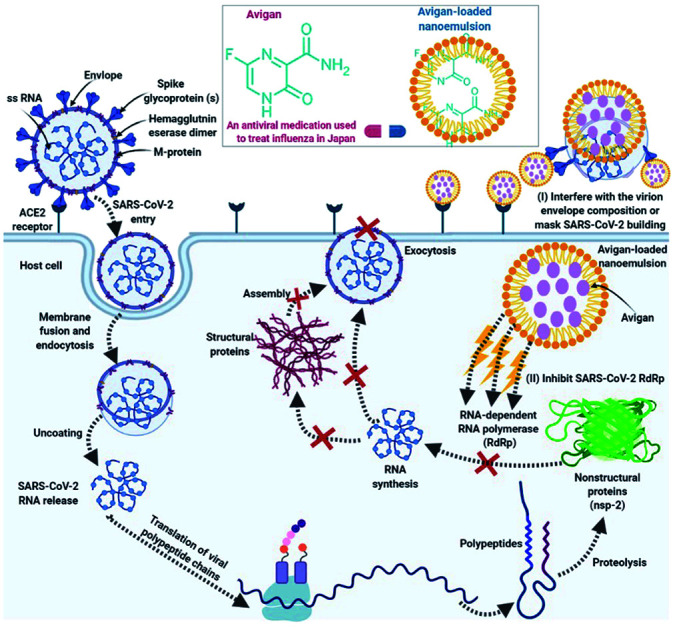

## Introduction

Severe Acute Respiratory Syndrome-Coronavirus-2 (SARS-CoV-2) has developed into a major pandemic and has contributed to the present surge in morbidity and mortality rates worldwide.^1^ Following the World Health Organization (WHO) declaration of SARS-COV-2 as a public health challenge of global concern,^2^ the international committee on Taxonomy of Viruses has classified SARS-COV-2 as the seventh member of the corona virus family associated with human infection. This classification follows previous outbreaks associated with Severe Acute Respiratory Syndrome-Coronavirus (SARS-CoV) in 2002 and Middle East Respiratory Syndrome-Coronavirus (MERS-CoV) in 2012.^3^ The original name of this new pathogen was 2019-novel coronavirus (2019-nCoV) as the pathogen associated with the infection. While, Coronavirus Disease-2019 (COVID-19) was first recommended in February 2020 by the WHO as Severe Acute Respiratory Syndrome-Coronavirus-2 (SARS-CoV-2) by the international committee on Taxonomy of Viruses.^4^

**Fig. 1:**
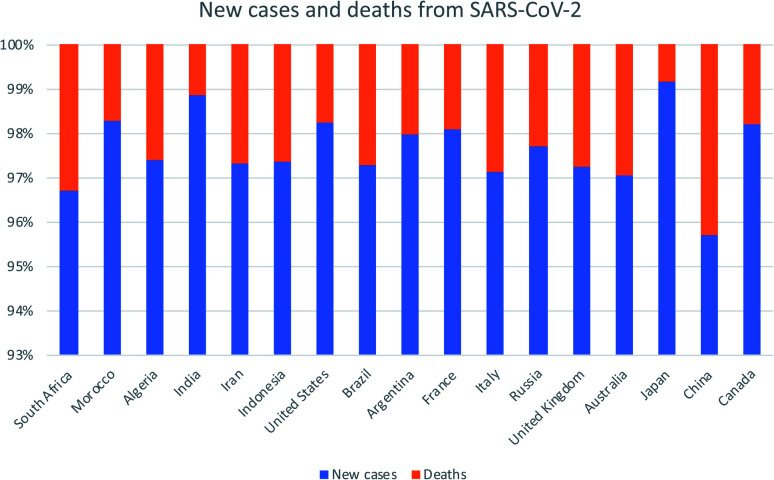
The global new cases and death cases due to SARS-CoV-2 as of 27^th^ May 2021. Adapted from European Centre for Disease Prevention and Control. Assessed on 27^th^ May 2021.

The SARS-CoV-2 outbreak has spread throughout the world;^5^ it is currently a threat of morbidity and mortality worldwide as shown in Fig. 1. One of the early cases of SARS-CoV-2 infection was traced to the seafood wholesale market in Wuhan, China, where different species of live animals are sold;^6^ this finding suggests that the virus was transmitted from animals to humans. Thereafter, reports of human-to-human transmission of the virus skyrocketed, as subsequent diagnosis of SARS-CoV-2 infection occurred among individuals who had no exposure to animals.^7^

## Etiology

Coronaviruses are enveloped, positive-sense single-stranded ribonucleic acid (RNA) viruses with a unique appearance resembling a solar corona due to projection of its characteristic club-like spikes. These viruses causes respiratory tract infections in humans, and are associated with enteritis in birds as well as a variety of diseases of pigs, bats, cows, dogs, cats, and chickens.^8,9^ SARS-CoV is a member of the group 2b beta coronaviruses. The Middle East Respiratory Syndrome-Coronavirus (MERS-CoV) was classified as within group 2c of beta corona viruses and is highly homologous to bat corona viruses HKU4 and HKU5 as documented in the literature.^10^

Infection with SARS-CoV-2 (also a beta coronavirus) follows a pattern that is similar to that reported for SARS-CoV and MERS-CoV.^11^ Previous studies revealed that SARS-CoV utilizes angiotensin converting enzyme 2 (ACE2) as a receptor for cell entry; this finding provided solid support of evidence suggesting that SARS-CoV originated in bats.^12^ By contrast, the cellular receptor used by the MERS-CoV is the enzyme dipeptidyl peptidase 4 (DPP4). Of note, MERS-CoV can only initiate infection *via* the use of species-restricted orthologs of DPP4, including those from humans, rabbits, bats, horses, and camels.^13^ Emerging evidence has confirmed that angiotensin converting enzyme 2 (ACE2) is also the host cellular receptor employed by SARS-CoV-2; this finding is not surprising given the nucleotide sequence homology reported in comparison between SARS-CoV-2 and SARS.^14–17^

Genomic sequencing has implicated either the Chinese (*Rhinolophus sinicus*) or the intermediate horseshoe bat (*Rhinolophus affinis*) as potential reservoirs of SARS-CoV-2; the source of the infection may be linked to an intermediate host. Identification of the intermediate wildlife host and reservoir may be critical as means to avoid future cross-species transmission of SARS-CoV-2; sequences isolated from the horseshoe bat as intermediate host were found to be similar to the sequence of SARS-CoV-2.^18,19^ Furthermore, viruses with ∼85–92% nucleotide homology to SARS-CoV-2 were identified in pangolins (family Manidae) during recent counter-smuggling activities in southern China; these results suggest that pangolins may be a potential intermediate host of SARS-CoV-2.^20^

The SARS-CoV-2 infection results in a wide spectrum of signs and symptoms, primarily those affecting the respiratory and gastrointestinal tracts. Moreover, circulating blood and specifically certain ABO blood groups have been implicated in differential susceptibility to SARS-CoV-2 and its complications. Zhao *et al.* (2020) reported that individuals of blood group O were somewhat less vulnerable to infection with SARS-CoV-2; those with blood group A demonstrated higher susceptibility than those with any of the other ABO blood groups; this has been attributed to the presence of natural serum anti-A antibodies.^19^ In one report, the complete blood count (CBC) of a female patient infected with SARS-CoV-2 after one week of hospitalization revealed an unusual leuko-erythroblastosis.^21^

Human-to-human transmission of SARS-CoV-2 is *via* droplets and through the respiratory tract, analogous to that reported for SARS-CoV and MERS-CoV.^22^ SARS-CoV-2 has also been detected on inanimate objects^23^ as well as in feces from infected patients^24^ which likely contributes in increasing in local transmission.

At this time, there are few specific therapeutic modalities established for the treatment of SARS-CoV-2;^25^ given the high economic loss and increasing number of infection, there is a dire need for more effective and safe therapeutic modalities. While several vaccine formulations are currently in various phases of testing, the daily rise in the number of confirmed cases worldwide suggests that more efforts are required. As such, this review shed light on unique nanomaterial-based drug delivery systems, which have already been successfully employed to deliver anticancer, antimicrobial, and antiviral drugs, might be employed to amplify efficiencies to anti-SARS-CoV-2 antiviral drugs.

## COVID-19 pathogenesis

The genome of Coronaviridae family viruses includes a 5′ cap structure and a 3′ poly-A tail with a genome length of 30–32 kb. Coronaviruses as a group have high genetic variability and recombination rates that facilitate generation of pathogenic and highly contagious strains.^26,27^

With emerging evidence, diverse variants of SARS-CoV-2 are now rampant. Among these new variants 501Y.V1 (B.1.1.7) of the United Kingdom lineage, 501Y.V2 (B.1.351) described in South Africa, 501Y.V3 (B.1.1.248) reported in Brazil, and B.1.4427 that was identified in California, USA. More recently, SARS-CoV-2 variants such as B.1.617 and B.1.618 were discovered in India by Indian scientists.^28,29^

Although the pathogenesis of SARS-CoV-2 infection remains poorly understood, the virus is genetically and structurally related to SARS-COV. As such, a focus on the mechanisms underlying MERS-CoV and SARS-CoV pathogenesis may elucidate critical factors associated with COVID-19.^30^ Among the pathological findings that have been elucidated, SARS-CoV-2 reaches the alveolar epithelial cells of the lower lung *via* transit through the respiratory tract, beginning with mucous membranes in the nasopharynx.^31,32^

The SARS-CoV-2 envelope spike glycoprotein can recognize and bind to the ACE2 receptor of lung alveoli with higher affinity than that associated with SARS-CoV; the virus then invades the host cell *via* clathrin-mediated endocytosis.^33^ As SARS-CoV enters the target cells, the viral genome discharged and is ultimately translated into one basic protein and two polyproteins in the cytoplasm. The viral genome then undergoes replication; and the newly formed envelope glycoproteins are combined with the nucleocapsid within the endoplasmic reticulum and Golgi, which results in vesicles containing virus fragments. These vesicles fuse with the plasma membrane in order to export virus progeny which can then infect neighboring cells and/or enter the circulation to invade other cells that express the ACE2 receptor.^34,35^

Viral infection also results in extensive immune and inflammatory reactions in the host which can lead to severe tissue damage. Acute respiratory distress syndrome (ARDS) is the most common immunopathological complication of COVID-19.^36^ The pathogenic mechanisms underlying ARDS are complex and difficult to control; a number of published reports have suggested that the ensuing cytokine storm plays a pivotal role in the pathogenesis of ARDS due to the large and seemingly uncontrolled release of pro-inflammatory cytokines including interferon (IFN)-α, IFN-γ, interleukin (IL)-1β, IL-6, IL-12, IL-18, IL-3, tumor necrosis factor (TNF)-α, transforming growth factor (TGF)-β, IL-2, IL-10, and IL-1RA, and by increasing the synthesis and release of chemokines, including CCL2, CCL3, CXCL8, CXCL9, and CXCL10.^30,37,38^

## Clinical presentation and diagnosis of COVID-19

The clinical spectrum of SARS-CoV-2 infection covers a wide range and varies from asymptomatic to the most severe manifestations involving hospitalization and respiratory support. The incubation period for COVID-19 is currently believed to be ≤14 days following exposure.^39^ Recent publications include reports of symptoms that vary from mild to severe. The most common clinical features associated with COVID-19 are fever, cough, severe dyspnea, sore throat, expectoration, muscle soreness, and fatigue; other symptoms noted less frequently are headache, hemoptysis, rhinorrhea, and gastrointestinal symptoms, including nausea, diarrhea, and vomiting.^40–42^

Among confirmed cases, patients with generally mild symptoms are likely to recover after an illness of approximately one week in duration while those with severe and critical findings may progress to respiratory failure, acute cardiac injury, acute renal injury, shock, and multiple organ dysfunction syndrome (MODS).^43^ The most comorbidities reported most frequently among those with severe disease include cardiovascular disease, diabetes mellitus, hypertension, lung disease, cancer, chronic kidney disease, and obesity.^43^

Clinical diagnosis of SARS-CoV-2 infection is based on epidemiological history, clinical presentation, the reverse-transcription polymerase chain reaction (RT-PCR) to detect virus-specific nucleic acid, computed tomography (CT) scan, blood cultures, and immunological testing for virus antigen or serum anti-SARS-CoV-2 antibodies.^30^

An RT-PCR test is used to confirm suspected cases of COVID-19; this method involving enzymatic detection of SARS-CoV-2 RNA in sputum, nasal and throat swabs, or in broncho alveolar lavage fluid.^44^ The gene targets of the RT-PCR tests include sequences encoding structural proteins as the nucleocapsid (N), envelope (E), and spike (S) glycoprotein; other targets include RNA-dependent RNA polymerase (RdRp) and regions in the first open reading frame.^45^ On occasion, SARS-CoV-2 can be detected in the peripheral blood and on rectal swabs despite negative throat swab tests; this result suggests that these patients can act as carriers of infection and confirms the importance of testing samples from different tissues and body sites in order to confirm infection.^46^ Due to the comparatively high false-negative rates reported for the virus RT-PCR test, many clinicians recommend performing a CT scan for those with negative RT-PCR screening in cases of high clinical suspicion as findings using this modality are typically more sensitive.^47^

The typical CT image associated with SARS-COV-2 infection includes ground-glass opacity with consolidative abnormalities in more severe cases with a bilateral peripheral distribution, primarily involving the lower lobes.^48,49^ There are other diagnostic techniques, including serologic tests that can detect antibodies to SARS-CoV-2; these tests will ultimately lead to the identification of patients with current infection and importantly, who have recovered from apriorSARS-CoV-2 infection.^30^

## Prevention of COVID-19 and surface disinfection

As there are few effective drugs or vaccines, the main strategies used to prevent SARS-CoV-2 infection focus on avoiding exposure to individuals who might be infected, blocking the route of transmission and providing protection for the highly-susceptible individuals.^50–52^

Fundamental standard measures include use of a face mask when in public, being careful to avoid touching one's face with unwashed hands, following routine respiratory hygiene practices, such as covering coughs and sneezes with tissues, frequent hand washing with soap and water for at least 20 seconds, utilizing hand sanitizer that contains at least 60% alcohol, and maintaining a distance from suspected or confirmed cases; furthermore, healthcare providers must use N95 respirator masks or a filtering face piece 2 (FFP2) while interacting with suspected or confirmed cases of COVID-19.^53^

The common prevailing challenges in biomedical science are the drug-resistance of some infectious viruses, output growth protocols, and their efficiency in courses of toxicity, remedial time, and side impacts on the individual cells. Additionally, discovering the infection-causing viral pathogen, monitoring the disease, and inhibition of some fatal diseases are important for public health.^54^

Human-to-human spread of SARS-CoV-2 takes place within incubation periods of between 2 and 14 days;^55^ transmission takes place *via* aerosol droplets and also *via* contaminated hands or surfaces. There may be other forms of transmission that have not been identified yet. Coronavirus pathogens, including SARS-CoV, MERS-CoV, and SARS-CoV-2, can persist on inanimate surfaces such as metal, glass, or plastic for more than 9 days.^56^ There is a varied range of disinfectant solutions that can be used to eliminate pathogens from surfaces.^57^ All surfaces that may have been in contact with virus pathogens should be fully-cleaned with water and disinfectants.^58^

A recent research study revealed that silver (Ag) NPs have excellent antiviral potential and may be an exceptional choice for future development.^59^ Nanofiber-based face respirators loaded with Ag NPs served as notably successful antimicrobial and antiviral disinfectants and may become the principal individual defensive agents that can be used to prevent the spread of the infection. Furthermore, there are a number of broad-based research projects in progress that focus on the use of nanomaterials as part of an immunization strategy for COVID-19. With respect to diagnostics, nanotechnology has been used extensively for the development of sensors that might accelerate the rate of current COVID-19 tests.^60^

Preliminary studies have also focused on nanomedicines. Ag NPs may have some advantages over the traditional biological solutions for cleaning and disinfection due to lower volatility; activities tested at even the lowest concentrations display low minimum inhibitory concentrations for virus pathogens.^59^ Moreover, Ag NPs may play a major role in bacterial, and viral disinfection due to their capacity to enrich for reactive oxygen species (ROS);^61^ for example, Ag NPs can effectively-generate extracellular ROS which could destroy influenza virus (IV) infection as an example.^62^ Intensive research is needed to verify these properties and to deploy these agents in the fight against COVID-19.

## Current medications used to treat COVID-19

Many research is focused on new drug development.^63^ Currently, various compounds and drugs are under clinical investigation for their efficacy in treating SARS-CoV-2. A recent study from Rosa *et al.*,^64^ highlighted 24 clinical trials that featured over 20 medications under exploration for the treatment of SARS-CoV-2; these can be classified into 8 main categories including (1) antiviral drugs that inhibit virus replication, including favipiravir (Avigan), remdesivir, umifenovir (Arbidol) and oseltamivir. (2) Immunomodulatory proteins such as interferons, (3) human antibodies including immune globulin (IVIG) and monoclonal bevacizumab (Avastin), (4) antiretroviral medications such as HIV-1 protease inhibitors, lopinavir and the CYP3A inhibitor, ritonavir (Norvir), (5) corticosteroids including methyl-prednisolone (Depo-Medrol), (6) bacterial-derived antibiotics such as azithromycin, (7) anti-parasite modalities, including ivermectin, and (8) traditional Chinese medicines (TCM). The complete details regarding the current medications used to treat COVID-19 are summarized in Table 1.

**Table 1:** Current medication used to treat COVID-19 disease, their molecular formula, treatment, reaction mechanism, and limitation

Medication	Molecular Formula	Chemical Formula	Treatment	Reaction mechanism	Limitation	Ref.
Favipiravir (Avigan), remdesivir, umifenovir (Arbidol) and oseltamivir	C_5_H_4_FN_3_O_2_, C_27_H_35_N_6_O_8_P, C_22_H_25_BrN_2_O_3_S, and C_16_H_28_N_2_O_4_, respectively	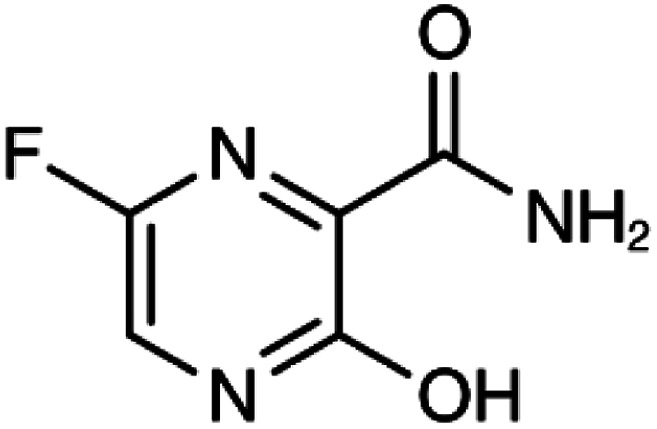 , 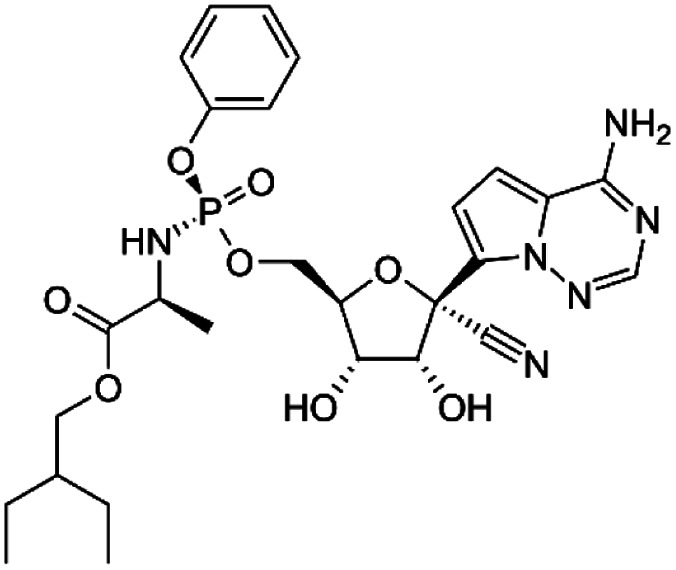 , 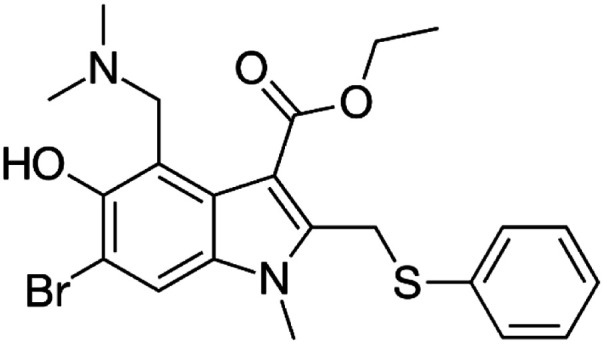 , and 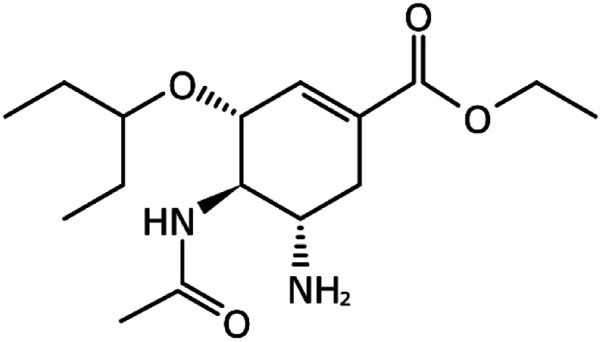 , respectively	Antiviral drugs that inhibit virus replication	Favipiravir (as example) binds to and inhibits RNA dependent-RNA polymerase (RdRp), which ultimately-prevents viral transcription and replication	Clearly, it would have limitations in its use for pregnant and potentially-pregnant women. Blinded, controlled trials are needed to establish whether it will be useful overall	65, 66
Interferons	Interferon-alfa-2B; C_16_H_17_C_l3_I_2_N_3_NaO_5_S	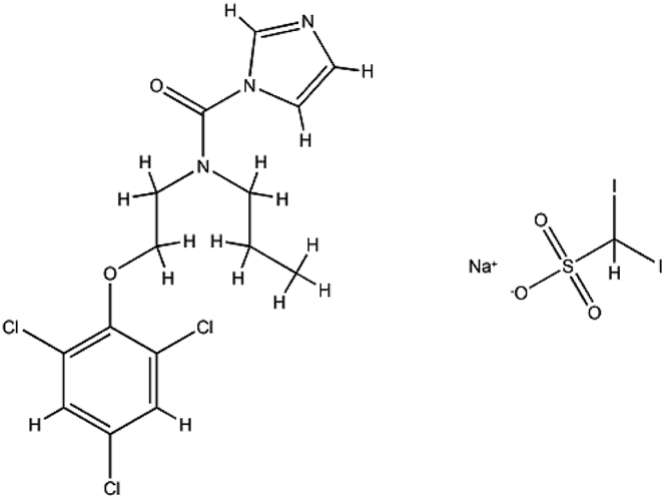	Immunomodulatory proteins made and released by host cells in response to the presence of several viruses	Interferon induces the expression of several hundred genes, names interferon-stimulated genes (ISGs) both in infected and neighboring cells. The products of the ISGs, in turn, allow the establishment of a so-called antiviral state, which is able to prevent, or at least limit, viral replication	The combination of interferon and ribavirin is now no longer used as safer, shorter highly-effective and more tolerable tablet only treatments are now available	67
Human antibodies including immune globulin (IVIG)	C_6332_H_9826_N_1692_O_1980_ S_42_	—	Immunoglobulin therapy, also known as normal human immunoglobulin, is the use of a mixture of antibodies to treat a number of health conditions	Intravenous immune globulin (“IVIG”) is a product made up of antibodies that can be given intravenously (through a vein). Antibodies are proteins that a human body makes to help the fight infections viruses	One reason you might need IVIG is if your body does not make enough antibodies	68
HIV-1 protease inhibitors (Indinavir sulfate)	C_36_H_49_N_5_O_8_S	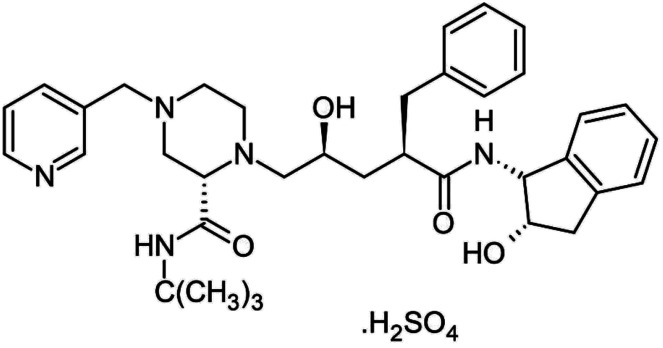	Antiretroviral medications used to treat HIV. The goal of these drugs is to reduce the amount of HIV virus in the body (called the viral load) to levels that are undetectable. This slows the progression of HIV and helps treat symptoms	Protease-mediated maturation of HIV-1 virus particles is essential for virus infectivity. Novel class of antiretroviral drug termed maturation inhibitors, which target cleavage sites. The exact mechanism is not clearly-understood, its substrate, and the chemical reaction of peptide bond cleavage had been detected	Unfortunately, most of the HIV-1 protease inhibitors are accompanied by side effects in long-term treatment	69
Corticosteroids-including methyl-prednisolone	C_22_H_30_O_5_	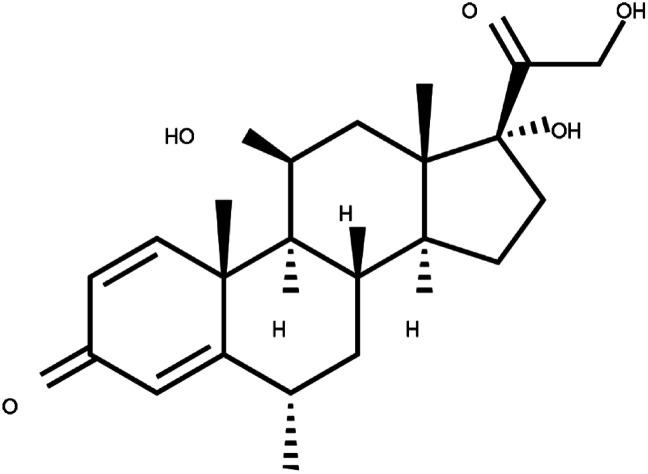	Clinical use of methylprednisolone is mainly due to its anti-inflammatory and immunosuppressive activity in the human body	Corticosteroid-resistant asthma is defined as a failure to improve FEV1 or PEF by over 15% after treatment with oral prednisolone 30–40 mg daily for 2 weeks	Corticosteroids including methylprednisolone can increase blood glucose, worsen pre-existing diabetes, and predispose those on long-term corticosteroid therapy to diabetes mellitus. Also, A course of broad-spectrum antibiotics must be achieved	70
Azithromycin	C_38_H_72_N_2_O_12_	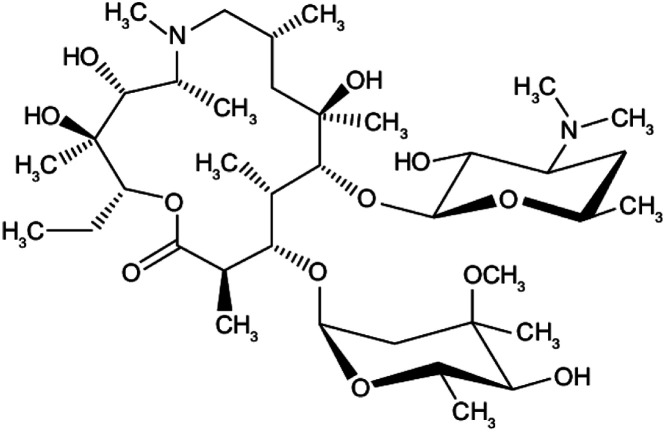	Bacterial-derived antibiotics used to treat Prevotella infections and decreases Prevotella-induced inflammation. Azithromycin has other attractive pharmacological and therapeutic properties in the search for COVID-19 drug therapy	Prevents bacteria from growing by interfering with their protein synthesis. It binds to the 50S subunit of the bacterial ribosome, thus inhibiting translation of mRNA. Azithromycin appears to decrease the COVID-19 virus entry into cells. In addition, it can enhance the immune response against viruses by several actions	There are mixed reports of effectiveness when azithromycin was used along with other medications to treat other viral respiratory infections	71
Ivermectin	C_47_H_72_O_14_	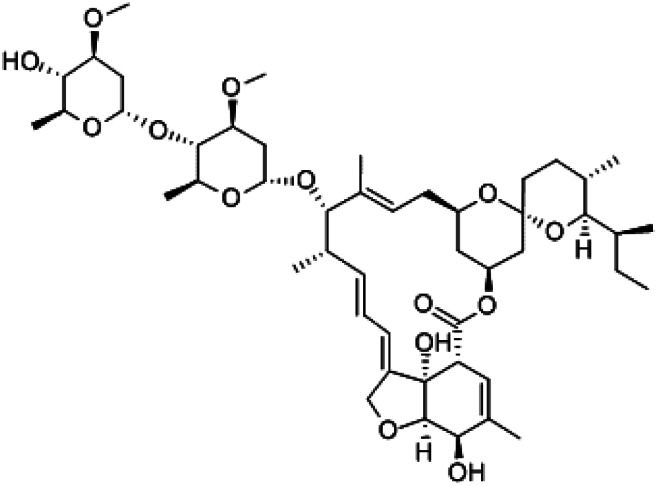	Anti-parasite modalities, including Ivermectin is an inhibitor of the SARS-CoV-2 *in vitro*. Ivermectin is FDA-approved for parasitic infections, and therefore has a potential for repurposing	Ivermectin binds to and destabilizes the Impα/β1 heterodimer thereby preventing Impα/β1 from binding to the viral protein (bottom) and preventing it from entering the nucleus. This likely results in reduced inhibition of the antiviral responses, leading to a normal, more efficient antiviral response. A single treatment able to effect ∼5000-fold reduction in virus at 48 h in cell culture	Since drugs that inhibit the enzyme CYP3A4 often also inhibit P-glycoprotein transport, the risk of increased absorption past the blood–brain barrier exists when ivermectin is administered along with other CYP3A4 inhibitors. These drugs include statins, HIV protease inhibitors, many calcium channel blockers, lidocaine, the benzodiazepines, and glucocorticoids such as dexamethasone	72

## Biomedical application of nanotechnology-based approaches in SARS-CoV-2 treatment

Since the last decade, the application of nanotechnology in the diagnosis, vaccine production, treatment and prevention of diseases have received excellent research attention.^73^ Food and Drug Administration (FDA) has approved nanocrystal formulations, polymeric-based nanoparticles such as polylactic-*co*-glycolic acid (PLGA) and polymeric micelles, as well as lipid-based nanoparticles like liposomes.^74^ More so, other nanoparticles such as inorganic, protein-based and metallic nanoparticles are in the pipeline of getting approval for their use in drug delivery.^75,76^

In providing a solution to the menace of this ravaging SARS-CoV-2, clinical trials are ongoing to investigate and evaluate antiviral drugs, vaccines, immunomodulatory drugs, and neutralizing antibodies. The mechanism of these antiviral drugs is similar to the life cycle and structure of the SARS-CoV-2. For instance, Lopinavir/Ritonavir and Ivermectin inhibits viral protease. Arbidol Hydrochloride, rhACES, Leronlimab, block the membrane fusion. Also, Favipiravir/Tocilizumab and Remdesivir inhibit RNA polymerase, while EIDD-2801 provides broad-spectrum antiviral effects.^74^

Regarding these vaccines, irrespective of the mechanism of action, their effectiveness and stability depend on the delivery medium. The JNJ-78436735 and ChAdOx1 nCoV-19 employs non-replicating adenovirus vectors, INO-4800 needs a portable device known as CELLECTRA and lipid particles are utilized by mRNA-1273.^74^ Based on these delivery platforms, using the FDA approved nanoparticles may offer excellent results in vaccine delivery and the delivery of antiviral drugs.

In a most recent article, Chakravarty and Vora (2021) described carbon-based, inorganic metallic-based, lipid-based, and polymeric-based as nanoparticles that have been successfully employed to deliver antiviral drugs.^77^ Interestingly, in furtherance of its application as a drug delivery vehicle, some of these nanoparticles exhibit antiviral properties.^74,78^ Their size and surface characteristics make them suitable and preferred candidates to be encapsulated and loaded with different antiviral drugs.^79^ Although many antiviral drugs are currently in use to treat SARS-CoV-2, at the same time, experts have recommended the introduction of nanoparticles to deliver these drugs. The novel severe acute respiratory syndrome coronavirus 2 (SARS-CoV-2) is similar to the size of these nanoparticles, which are in nano-size of 60 to 140 nm. Hence, loading these antiviral drugs with nanoparticles may provide effective therapeutic outcomes.^80^ Most recently, dexamethasone nano-formulations, with inhalation and intravenous as the routes of administration to hyper-activated immune cells, have been suggested in the treatment of SARS-CoV-2.^81^

Emerging shreds of evidence have shown that comorbidity that comes with immunosuppression aggravate SARS-CoV-2 infection. Such an example is in patients undergoing immunotherapy.^82^ For this purpose, metallic and other two-dimensional nanomaterials will be a helpful platform to load these therapeutic agents to and deliver.

There are various classes of these two-dimensional nanomaterials available to deliver these antiviral drugs and vaccines for better therapeutic outcome in immunotherapy. Recently, these nanomaterials can be used as a medium to deliver different therapeutic agents such as photodynamic therapy (PDT), photothermal therapy and drug delivery. Chen and Colleagues (2020) synthesised a nontoxic, very stable nanocomposite made of tellurium–selenium (TeSe)-derived lateral heterojunction with a uniform size. The results of their study show accumulation of the formulated nanocomposite in the lung of the mice, tumour suppression, and induction of cancer cell apoptosis. More so, TeSe eradicated hepatocellular carcinoma and lung cancer in preclinical trials. Hence, the authors established the importance of TeSe nanocomposite as a PTT-based candidate to eliminate cancer.^83^ In another study in experimental mice, the synergistic effects of black phosphorus (BP)-based photothermal therapy loaded with anti-CD47 antibody-based immunotherapy was observed. Their findings demonstrated that a-CD47-BP-based photothermal therapy conjugate elicits local and systemic anticancer immune responses by activating adaptive and innate immunities.^84^

In recent times, silicon and black phosphorus represent some of the widely investigated inorganic nanomaterials for their biomedical applications, photothermal therapy, theranostics, drug delivery system and bio-imaging. Their ability in a wide range of applications has been attributed to the high drug loading capacity, biocompatibility, definite clearance pathways, less cytotoxicity, biodegradability, and excellent physicochemical properties.^85^ Phosphorus, as the core component of black phosphorus, is crucial to acid–base balance, important in energy metabolism and in the transfer of genetic materials. Hence, a coagent reason for the broad application of phosphorus and phosphorous-derived inorganic/two-dimensional nanoparticles in biomedical research.^86^

Furthermore, other inorganic nanomaterials such as gold nanorods, up conversion nanoparticle, black phosphorus nanosheets, MnO_2_, Mxene, single-walled carbon nanotubes, and graphene have been employed in photothermal therapy.^87^ Luo and Coworker have described black phosphorus as an alternative effective photothermal agent in cancer treatment owing to its high drug loading ability, excellent treatment effectiveness, and negligible cytotoxicity.^88^ A recent study utilized bath sonication and probe sonication to synthesized black phosphorus quantum dots and examined the photothermal effects of the nanocomposite fabricated on the C6 and MCF7 cancer. The result of this investigation revealed effective photothermal performance leading to stability in physiological medium, notable cell death and without cytotoxicity.^89^

To combat the ravaging SARS-CoV-2 pandemic, conjugating the currently available SARS-CoV-2 drugs with the FDA approved nanoparticles, inorganic nanoparticles, two-dimensional nanoparticles, biocompatible and biodegradable nanoparticles would be of great interest.

## Nanomaterial-based drug delivery systems

### Preparation of nanomaterial-based drug delivery systems

**Fig. 2:**
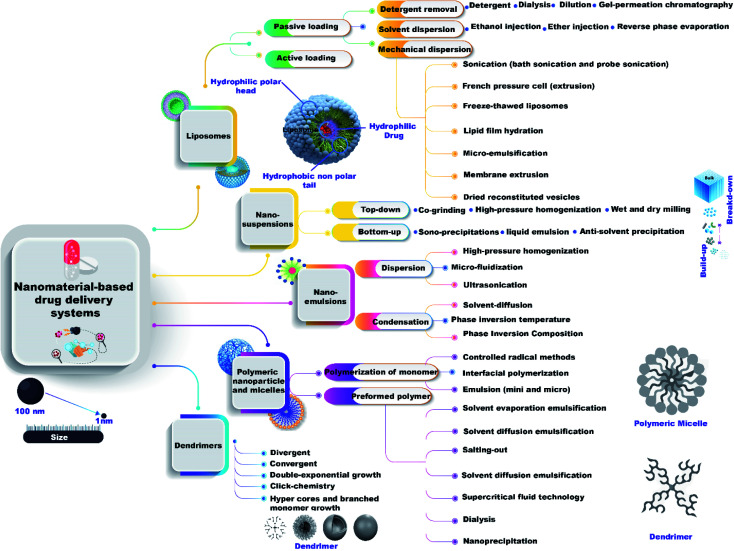
Different preparation methods of nanomaterial-based drug delivery systems.

There are many methods to prepare nanomaterial-based drug delivery systems that can be divided into two main approaches, bottom-up and top-down (Fig. 2) as the following:

#### Nano-emulsions

These can be prepared by two main methods, high-energy emulsification (dispersion) and low-energy emulsification (condensation). High-energy emulsification can be subdivided into three categories, high-pressure homogenization (HPH), micro-fluidization, and ultra-sonication. While, low-energy emulsification can be subdivided into solvent-diffusion, phase inversion temperature (PIT), and Phase Inversion Composition (PIC).^90^

#### Liposomes

These can be prepared by various methods that can be categorized into passive loading techniques and active loading techniques.^91^ Passive loading techniques have three types, mechanical dispersion, solvent dispersion and detergent removal methods (non-encapsulated material removal).

Mechanical dispersion methods are divided into 7 classes, sonication (bath sonication and probe sonication), French pressure cell (extrusion), freeze-thawed liposomes, lipid film hydration (by hand shaking, non-hand shaking or freeze drying), micro-emulsification, membrane extrusion, and dried reconstituted vesicles.

Solvent dispersion method has three types (ether injection, ethanol injection, and reverse phase evaporation method). Detergent removal methods have four types (dialysis, detergent, gel-permeation chromatography, and dilution).

#### Polymeric nanoparticles and micelles

These can be prepared by two main routes, polymerization of monomers methods and preformed polymer methods. Polymerization of monomers methods have many types such as emulsion (mini and micro), interfacial polymerization, and controlled radical methods. While, preformed polymer methods can be divided into solvent evaporation emulsification, solvent diffusion emulsification, salting-out, nano-precipitation, dialysis, and supercritical fluid technology.^92^

#### Dendrimer

These can be prepared by many methods such as divergent and convergent synthesis, double-exponential growth, click-chemistry, and hyper cores and branched monomer growth methods.^93^

#### Nano-suspensions

These can be synthesized by top-down methods including (wet and dry milling, co-grinding, and high-pressure homogenization) and bottom-up methods such as (anti-solvent precipitation, liquid emulsion, and sono-precipitations).^94^

### Unique advantages of nanomaterial-based drug delivery systems, their drug loading capacity, and the main differences for treating COVID-19

Due to the unique physicochemical properties of nanomaterials, their use in drug-delivery systems allows many superior advantages over the traditional routes for treating COVID-19 as the following

#### Nano-emulsions

Nano-emulsions are not toxic in nature, and do not cause irritation.^95^ In addition, they have the ability to enhance the bioavailability of drugs.^96^ They have physical stability and can help in solubilizing lipophilic drugs.^97^ Moreover, nano-emulsions possess tiny droplets with high surface area providing greater absorption capacity.^98^ They can be also formulated in different formulations including liquids, creams, foams, and sprays.^99^ Finally, they can be employed substitutes for liposomes and vehicles.^100^

For the drug loading capacity, Huil Gaoe *et al.*^101^ succeeded in synthesizing docetaxel-loaded nano-emulsion with a particle size of 72.3 nm. The average zeta potential was calculated to be −6.38 mV, the encapsulation performance was 93.1%, and the drug loading capacity had been assessed to be 2.87%.

Very few investigations have shown the mode of action of nano-emulsion of some essential oils on some deadly viruses.^102^ Most of the scientific papers prophesied that nano-emulsion of some essential oils could interfere with the virion envelope construction or mask the virus (COVID-19) building, which finally prevents COVID-19 adsorption and invasion into the host cells.^102,103^

#### Liposomes

Liposomes are cell-like lipid vesicles have an ordered phospholipid bilayer and possess a lot of advantages such as non-toxic, non-immunogenic, changing drug distribution *in vivo*, allowing sustained-release of drugs, elongating drug action time, enhancing drug treatment index, minimizing drug side effects, and entrapment of hydrophilic, ionic molecule, sand hydrophobic drugs.^104^ By adjusting the lipid materials, their particle size and surface chemistry can be controlled. Cationic liposomes (positively charged) can be employed in dose-dependent cytotoxicity and inflammatory responses.^105^

Regarding drug loading capacity, Zhi Wang *et al.*,^106^ synthesized paclitaxel-ss-lysophospholipid (paclitaxel-liposome) with an average diameter of 234.9 nm and good stability (zeta potential of −29.1 mV), the highest paclitaxel loading capacity determined was about 7.97%.

It was reported that, liposomes (positively charged) possess elevated potential for mucosal vaccinations due to the retention in the nasal cavity (negatively charged) causing a powerful immune effect,^107^ pointing to the creation of greater levels of immunoglobulins to fight COVID-19.^108^

#### Polymeric nanoparticles and micelles

Polymeric nanoparticles have two main categories (non-biodegradable and biodegradable materials).^109^ These polymers show no teratogenicity, non-toxicity, and biocompatibility. Their degraded materials in cells have no toxic effect.^110^ They are stable with many drugs. Self-assembled polymers can be used to intercept insoluble drugs. Allow controlled release of drugs because of their stable structure.^111^

For the drug loading capacity, Ilaria Fratoddi *et al.*,^112^ prepared polymeric NPs bio-conjugated with dexamethasone (DXM) by a changed surfactant free emulsion system. The loading capacity was investigated as a function of various functionality standards of the copolymer and varying quantities of DXM drug, reaching up to 90% of DXM loading for the obtained polymeric NPs.

Polymer nanoparticles composed of chitosan (Cs) drew special attention for intranasal treatment because of their non-toxic characteristics, biocompatibility, ability to open up strong linkages with epithelial cells,^113^ and strength to be changed into fancied appearance and sizes.^114^ Simultaneous incorporation with therapeutic composites, Cs can improve assertiveness of polymeric NP in mucosal conditions and the entrance to the mucosal membrane to resist any COVID-19 infection in the mucosal environment.^108^

#### Dendrimers

They are various-shaped, branched synthetic drug delivery systems that usually used for the administration of insoluble drugs.^115^ Their molecular weight can be tuned.^116^ In addition, they can be conjugated with various surface functional groups making them a unique drug delivery material.^117^ Due to their outstanding biological characteristics, they are widely used in biomedical and pharmaceutical applications.

Regarding the drug loading capacity, Fei Wang *et al.*,^118^ prepared poly-propylenimine (PPI) dendrimers, and the acetylated PPI dendrimers with differing degrees of acetylation varying from 14.2% to 94.3% used to encapsulate drugs such as methotrexate sodium. The concentrations of methotrexate sodium in dendrimer/methotrexate complex raised linearly with the proportion of acetylation of PPI dendrimer up to 88.9%, and the drug loading capacity gave 1.2 mg ml^−1^.

With the significant interactions with some viruses, dendrimers exhibited improved antiviral potential, limiting the host's disease. So, they became a major candidate in managing viral conditions like HIV, COVID-19, and influenza virus disease.^108,119^ Chahal *et al.*,^120^ investigated dendrimer NP encapsulating an antigen-expressing replicon mRNA and allowed vital CD8+ T-cell and antibody responses to defend against dangerous pathogens, such as Ebola, and H1N1 influenza. The principal findings can be tested against COVID-19 and such impact can diminish the destructive attack of COVID-19.^108^

#### Nano-suspensions

They are heterogeneous nano-sized dispersions of surfactant-stabilized insoluble drug particles.^94^ They possess good target-receptor binding. Allow excellent dissolution rate and bioavailability due to their large surface area and tiny size.^121^ They can be used when drugs are unable to form salt or having large molecular weight, dose, and melting point limiting the development of suitable formulations.^121^ Nano-suspensions can overcome the problems due to active pharmaceutical ingredients (API) by retaining it in a crystalline state enabling them with increased drug loading during formulation development. Allow accommodating large drug amount with reduced dose volume. Finally, they have large stability, allow drug sustained release, high efficacy *via* tissue targeting, minimum first pass metabolism, and deep lung deposition.^122^

For the drug loading capacity, Jingyi Hong *et al.*,^123^ synthesized annonaceous acetogenins (ACGs) nano-suspensions possessing an average particle size of 144.4 nm, a zeta potential measured to be (−22.9 mV) and an extraordinary drug loading capacity of 46.17%.

Nano-suspensions can significantly inhibit the virus inside the host body and block the virus entry into the host cells.^124^ Due to their high surface area and the capacity to adhere multiple antigens and composites on their surface. In addition, nanomaterials such as gold NPs and carbon quantum dots (CQDs) were described as encouraging agents for interaction with COVID-19 and blocking their entry into host cells.^108,124^

### Toxicity of nanomaterial-based drug delivery systems, with a risk analysis for therapeutic applications, and the animal experiments relating COVID-19

Due to the encouraging uses of nanomaterials-based drug delivery system, their toxicity to host cell and their risk analysis for therapeutic applications must be assessed as well as animal experiments relating COVID-19 the as the following:

#### Nano-emulsions

The nano-emulsions were tested for *in vitro* cytotoxicity utilizing SK-N-SH cell line and nasal ciliotoxicity investigation.^125^ The developed nano-emulsions did not show any toxicity and were reliable for intranasal drug delivery for brain tissue targeting. *In vitro* distribution investigations concluded that nano-emulsions had a distinguished release related to the drug solution.^126^

Regarding risk analysis for therapeutic applications, a fitting choice of ingredients is vital for an effective nano-emulsion formulation. Low-molar-volume oils are better than high-molar-volume oils, as they regularly confer more proper drug solubilization.^127^ New, innovative semisynthetic medium-chain derivatives, described as amphiphilic aggregates with surfactant characteristics, are favored. Consideration should be adjusted to the tolerability of the constituting excipients. Accordingly, recent attempts have concentrated on reducing the toxicity or inflammation of the nano-emulsion formulations. The influence of the surfactant/co-surfactant weight ratio in the nano-emulsion formulation must be considered. It is reasonable to obtain acceptable features by suitably modifying the level of surfactants, oil, and secondary surfactants to avoid their risk in therapy applications.^127^

#### Liposomes

Phospholipids are the principal ingredients of most utmost liposomes. An extended examination of these naturally occurring mixtures recommended them to be incredibly reliable for pharmaceutical application.^128^ Olsen *et al.*,^129^ synthesized Adriamycin encapsulated in liposomes and studied their toxicity *in vitro* (chick heart cells) and *in vivo* (5-fold reduction in LD_50_ in mice). As compared with free Adriamycin, liposome-encapsulated Adriamycin was less toxic *in vitro* toward chick heart cells. Adriamycin showed short-term (from 4 to 14 days) and long-term (from 45 to 70 days) toxicity in mice. Liposome-encapsulating Adriamycin improved both short-term (from 20 to 50 mg kg^−1^) and long-term (from 10–15 mg kg^−1^ to 25–30 mg kg^−1^) LD_50_ levels.

For risk analysis, liposome particle size and drug encapsulation performance are two essential product features, and knowledge of the potential risks are critical. To accomplish this, two cause-and-effect diagrams (Ishikawa diagram) were created to recognize the possible causes for product variability. Following the risk analysis, the succeeding eight variables were classified as high-risk parts influencing liposome; drug encapsulation and particle size: lipid concentration, drug concentration, cholesterol concentration, buffer concentration, hydration time, sonication period, number of freeze–thaw cycles, and extrusion pressure.^130^

Pooladanda *et al.*^131^ synthesized iRGD conjugated nimbolide liposomes (iRGD-NIMLip) for treating COVID-19 related diseases and studied their effect *in vivo* (male C57BL/6 mice (5–6 weeks old)). The animal experiment assumed that iRGD-conjugated nimbolide liposomes treatment significantly repressed oxidative stress and cytokine storm associated with free-drug and showed higher activity than dexamethasone (DEX). The synthesized iRGD-NIMLip cancelled the LPS induced p65 NF-κB, Akt, MAPK, Integrin β3 and β5, STAT3, and DNMT1 expression. Finally, iRGD-NIMLip could be a hopeful novel drug delivery system to target critical pathological values recognized in COVID-19 associated cytokine storm.

#### Polymeric nanoparticles and micelles

Polymeric NPs (like polybutylcyanoacrylate NPs; PBCA NPs) are applicants for drug delivery operations, passing through the blood–brain barrier (BBB), so their toxicity must be examined. Cells were exposed to PBCA NPs *in vitro* and *in vivo* for observing their life and death behavior.^132^ Viabilities of HeLa and HEK293 cells following NP treatment were quantified by analyzing the cellular metabolic action (MTT-test).^133^*In vivo* tests did not reveal any polymeric NP-induced neuronal destruction.^132^ For risk analysis, polymeric NPs were generated based on insufficient laboratory data sets to foretell the bio-distribution and removal kinetics of polymeric NPs with a relatively restricted set of physicochemical modifications (like size and surface charge). These modified polymeric NPs are intended to support researchers choosing optimal NPs features for additional growth or risk evaluation analysis.^134^

#### Dendrimers

Dendrimers can decrease some organ toxicity regarding solubilized cancer drugs given by intraperitoneal injection.^135^ 6-Mercaptopurine was FDA approved anticancer drugs, which are identified as hepato-toxins.^136^ The solubility of 6-mercaptopurine can be improved by combining them with a dendrimer.^137^ The alanine transaminase (ALT) enzyme level was tracked to examine liver destruction.^138^ After the dendrimer encapsulates 6-mercaptopurine, a notable decrease in hepatotoxicity was recognized, and ALT level from the protected groups (6-mercaptopurine + dendrimer) was 36%, which is lower than 6-mercaptopurine only.^135^

Concerning the risk analysis, the sensitivity of the dendrimer structure to *in vivo* biodegradation may also affect the rate of dendrimer removal from the organization. Still, exterior functionalization with non-biologic collections (like non-natural amino acids) reduces dendrimer building disruption.^139^ The method suggested examining the surface adsorption of a collection of small-molecule inquiries and making a ‘surface adsorption index’ to foretell the binding of bio-molecules which represents a vital function in managing the bio-distribution behavior of dendrimers.^140^ After this, researchers have done physiologically based pharmacokinetic models to assume the mass-time bio-distribution outlines for various dendrimer.

Khaitov *et al.*,^141^ synthesized the qualified siRNA-peptide dendrimer (siR-7-EM/KK-46) formulation for treating COVID-19 related diseases and studied their effect *in vivo* (The Syrian hamsters (females, 4–5 weeks of age, 40–60 g weight)). The animal experiment assumed a meaningful decline of virus titer and lung inflammation in animals presented to the inhalation of siR-7-EM/KK-46 *in vivo*. Thus, they formed a therapeutic approach for COVID-19 based on the breath of a qualified siRNA-peptide dendrimer formulation.

#### Nano-suspensions

Nano-suspensions have demonstrated their efficacy in drug delivery with water- and lipid-insoluble drugs.^142^ They are outstanding because of their integrity and most reliable over other application procedures. In addition, they improve the dissolution speed and saturation solubility of the drugs.^143^ For example, fucoxanthin is insoluble in water and sparingly soluble in acetone and possesses an excellent thermostability and stability at a wide pH range. Acute toxicity was examined (LD_50_; 2000 mg kg^−1^) and was found to be non-toxic.^144^ These high doses of fucoxanthin extract and solubility characteristics are obstacles in the traditional drug delivery. However, nano-suspensions composites could easily overcome these limitations.^144^

## Avigan as an example of COVID-19 current drugs

Biomedical experts in China announced promising results from a Japanese influenza drug that seemed to be effective for cases of SARS-CoV-2.^145^. Zhang Xinmin or China's science and technology ministry, reported overall promising results with favipiravir (the generic name of Avigan, produced by a subsidiary of Fujifilm) in 350 clinical cases tested in both Shenzhen and Wuhan.^146^

Avigan is the trademarked name of the antiviral drug, favipiravir.^147^ This drug was first produced by Fujifilm Toyama Chemical Company and licensed in 2014 for medical use in Japan.^148^ It was particularly-recommended for the treatment of specific forms of influenza that were not adequately covered by other antiviral or vaccines.^149^ In addition to its role as an antiviral drug for the treatment of influenza, it is believed to have impact on several other viral diseases; its chemical formula is a pyrazinecarboxamide derivative and as such is similar in structure to other antiviral compounds, including T-1106 and T-1105.^150^

Favipiravir was registered for the treatment of new influenza (*i.e.*, strains that are exceptionally-difficult to treat); this did not include routine seasonal influenza.^151^ In February 2020, the role of favipiravir as an agent for the treatment of SARS-CoV-2 infection was explored in China and in Japan for the treatment of new COVID-19 cases.^152^

With respect to the history of this drug compound, it was first recommended in China in 2019 for the treatment of clinically-significant coronavirus disease. Italy accepted the drug in March 2020 with the intent to perform clinical trials in three areas that were hard-hit by COVID-19.^153^ This drug is currently under consideration (April 2020) for three clinical projects to be carried out in the USA.

The mechanism of action underlying drug therapy with respect to COVID-19 is linked to its capacity to interfere with the activity of the viral enzyme RNA-dependent RNA polymerase;^154^ as such, favipiravir is believed to act by preventing virus replication inside the host cell.^155^ Other investigations reported that treatment with favipiravir results in fatal mutations during viral RNA transcription, which would lead to a reduction in the viable viral progeny.^156^ Favipiravir did not hinder host DNA and/or RNA organization in the mammalian cells and thus it is presumed to be safe for use and without any specific toxicity.^157^

Favipiravir is metabolized inside the host cell and converted to active favipiravir-ribofuranosyl-5′-triphosphate (favipiravir-RTP). The human enzyme hypoxanthine-guanine phosphoribosyl transferase (HGPRT) is responsible for activates favipiravir.^158^

Favipiravir is available for human consumption in the form of tablets (oral usage) or as a pro-drug that can be used for intravenous infusion.^159^ Scientists in Japan are currently using this drug in clinical investigations on coronavirus cases in an effort to reduce disease severity, as it may prevent ongoing SARS-CoV-2 replication in infected patients.^160^

Patients infected with COVID-19 in China who underwent treatment with Avigan became SARS-CoV-2 negative after a mean of 5 days.^161^ Radiographic studies revealed improvements with respect to lung findings in ∼90% of patients treated with favipiravir, compared to only 65% of those who did not receive drug treatment.^162^ Currently, use of favipiravir for COVID-19 patients requires administrative permission because it was designed and evaluated in patients infected with influenza.

Nanotechnology can foster the development of effective medications for COVID-19 either by acting as drug delivery carriers or by their high antimicrobial and antiviral abilities as shown in our previously-published article.^163^ The next section presents an example of a nano-drug composed of Avigan-encapsulated nanoemulsions.

## Avigan-loaded nano-emulsions as example of nanodrugs

With increasing concerns regarding the number and severity of adverse events, recent studies have focused on new avenues and modalities for drug delivery. Toward this end, we propose a consideration of Avigan-loaded nano-emulsions as a promising nano-based delivery system for the treatment of COVID-19.

**Fig. 3:**
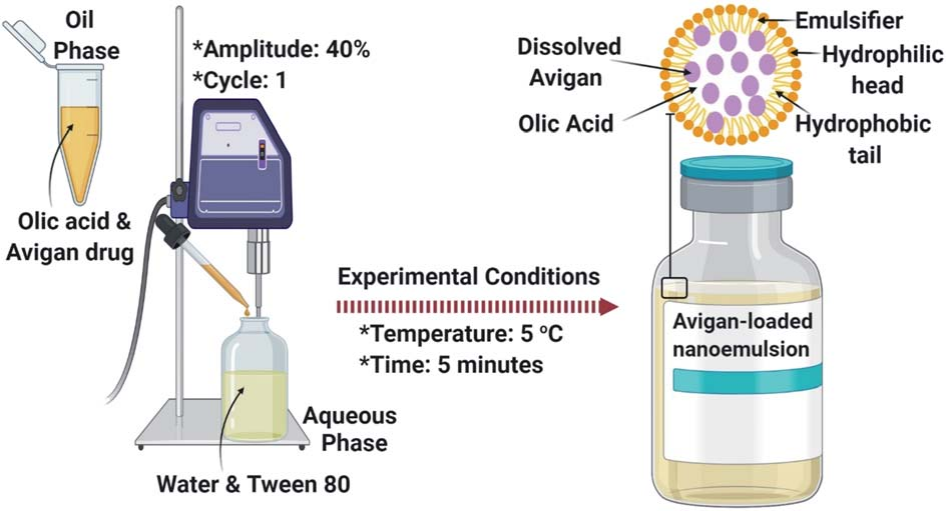
The process of preparing Avigan-loaded nano-emulsions.

Favipiravir is synthesized as a white to bright yellow powder.^164,165^ It is only marginally soluble in acetonitrile and methanol and slightly soluble in water and absolute ethanol. Also, favipiravir is slightly soluble in water buffered at pH 2 to 5.5 and sparingly soluble at pH 5.5 to 6.1.^166^ As such, favipiravir may be difficult to administer due to its limited solubility in water solubility. Drug solubility patterns have a direct impact on its pharmacokinetics, speed, and degree of absorption, bioavailability, and therapeutic potency; a more suitable therapeutic formulation will be required.^167^ As such, we present favipiravir-encapsulated nano-emulsions as promising drug delivery carriers for patients with COVID-19 as displayed in Fig. 3.

Nano-emulsions are kinetically-permanent and are dispersed in biphasic form as oil-in-water which is stabilized by an interfacial layer that includes an amphiphilic surfactant (Tween 80).^168^ Nano-emulsion formulations include submicron-sized particles that are typically 5 nm to 200 nm in diameter. Nano-emulsions present many possible benefits with respect to oral administration of active pharmaceutical constituents, including effective encapsulation of slightly water-soluble drugs such as favipiravir; they promote a strong balance vis-à-vis disconnection, coalescence, and flocculation. In addition, nano-emulsions can promote drug bioavailability and penetration.^169^

By encapsulating favipiravir inside the synthesized nano-emulsions, we can confirm that the slightly soluble drug will be centered in the core of the nano-emulsions and will be capable of binding by weak van der Waals forces^170^ as shown in Fig. 3. This will facilitate binding to the virus RNA-dependent RNA polymerase after its release from the nano-emulsions, and subsequently hinders virus replication through inhibiting the SARS-COV-2 RNA replication.

## Proposed action mechanism

The mechanisms of action associated with the efficacy of nano-emulsions for the treatment of COVID-19 can be classified into two main categories:

(I) Nano-emulsions have the capacity to interfere with the virion envelope composition or mask the virus (SARS-CoV-2) building, which finally prevents SARS-CoV-2 adsorption and invasion into the host cells.^102^

(II) Inside host cells, favipiravir carried by the nano-emulsion can interfere with the actions of the SARS-CoV-2 RNA-dependent RNA polymerase (RdRp).^171^

**Fig. 4:**
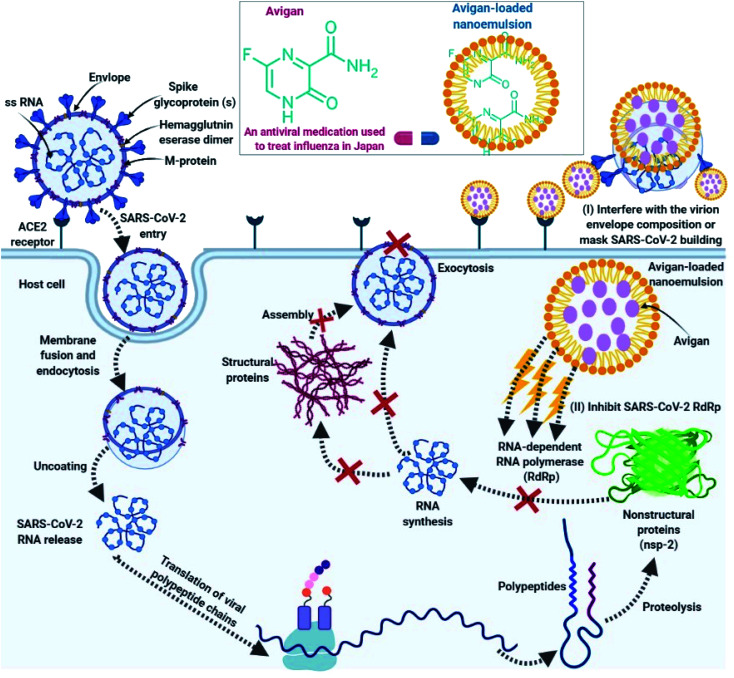
Mode of action of Avigan-loaded nano-emulsions towards SARS-CoV-2 outside host cell (I) interfere with the virion envelope composition or mask the virus (SARS-CoV-2) building, so prevents SARS-CoV-2 adsorption and invasion into the host cells by Avigan-loaded nano-emulsion and (II) actions inside host cell including, interference with the viral enzyme (RNA-dependent RNA polymerase).

As such, we anticipate that favipiravir-loaded nano-emulsions could be capable of effectively-preventing SARS-CoV-2 adsorption and destroying its structure. This will prevent SARS-CoV-2 from surface-mediated attachment to the host cell as shown in Fig. 4.

Second, if the virus does in fact enter the host cell, favipiravir-loaded nano-emulsions will be able to interact with the viral polymerase (RNA-dependent RNA polymerase; Fig. 4).^154^ Favipiravir acts to prevent replication of the virus inside the host cell^155^ and generates fatal mutations during viral RNA transcription, thereby decreasing the population of viable viral progeny.^156^

## Nanomaterial-based drug delivery systems as nanovaccines

Nanomedicine is a new and rapidly developing area, and the synthesized NPs can be applied in drug formulations due to their pharmacokinetics, security, efficiency, and targeting capability.^76^ Numerous drug-based NPs have been recorded in clinical cases. However, drug-based NPs meet a lot of difficulties because of their toxicity problems, demand for better validation, and lack of administrative guidelines.^76^ Nanotechnology has joined with vaccine progress in the fight toward infections produced by bacterial or viral diseases, as well as cancerous tumors, appearing in nanovaccines, which are important.^172^ Nanovaccines have many benefits over conventional vaccines as antigens can be encapsulated in nanocarriers to withdraw antigenic degeneration. Also, antigen-presenting cells (APCs) can immediately prepare and phagocytize nanovaccines.^173^

Nanomaterial-based drug delivery systems have afforded one-of-a-kind possibilities to improve the therapeutic efficiency of cancer and SARS-CoV-2 vaccines (antigens), while molecular or nano-adjuvants and nano-carriers are usually applied in nanovaccines.^174^ In melanoma, colon cancer, and human papillomavirus E6/E7, nano-vaccines have caused significant immune responses that inhibited tumor growth.^175^ COVID-19 vaccines have been produced unprecedentedly, which would not have been possible without decades of fundamental research on delivery nanotechnology. Lipid-based nanoparticles have performed a crucial part in the progress of COVID-19 vaccines have therefore been regarded as the frontrunner in nanoscale drug delivery systems.^176^

Vaccination represents the most reliable line of defense against infectious disorders and is essential in reducing the pandemic extent of developing pathogens to which a population has insufficient immunity.^177^ In current years, mRNA vaccines have been introduced as the original frontier in vaccination, owing to their fast and straightforward growth while giving a safer alternative to conventional vaccine technologies such as live or attenuated viruses (SARS-CoV-2).^178^ Recent discoveries in mRNA vaccination have been through formulation with lipid nanoparticles (LNPs), which present both security and improving the delivery of mRNA vaccines *in vivo*.^179^ With the recent success of mRNA vaccines developed by Moderna and BioNTech/Pfizer against COVID-19, mRNA technology and lipid nanoparticles (LNP) have never received more attention.^180^ Emily H. Pilkington *et al.*,^181^ displays the most excellent mRNA-LNP vaccines that have just been accepted for emergency treatment and are in clinical cases, with a focus on the unusual construction of several COVID-19 vaccines, quicker than any other vaccine in history. Another study crated by Mai N. Vu *et al.*,^182^ stated that hemagglutinin functionalized liposomal vaccines improve the germinal center and follicular helper T cell immunity, which in turn obtains a solution of COVID- 19 invasion.

A key concept in nanomedicine is encapsulating therapeutic or diagnostic agents inside nanoparticles to prolong blood circulation time and to enhance interactions with targeted cells.^183^ During circulation and depending on the selected application (*e.g.*, cancer drug delivery or immune modulators), NPs are required to possess low or high interactions with cells in human blood and blood vessels to minimize side effects or maximize delivery efficiency.^184,185^ Here, the first comprehensive method to analyze cellular interactions of both synthetic and commercially available NPs under human blood flow conditions in a microvascular network is developed. Importantly, this method allows to unravel the complex interplay of size, charge, and type of NPs on their cellular associations under the dynamic flow of human blood. This method offers a unique platform to study complex interactions of any type of NPs in human blood flow conditions and serves as a useful guideline for the rational design of liposomes and polymer NPs for diverse applications in nanomedicine.^184^

Poly ethylene glycol (PEG) is widely used as a gold standard in bio-conjugation and nanomedicine to prolong blood circulation time and improve drug efficacy. The conjugation of PEG to proteins, peptides, oligonucleotides (DNA, small interfering RNA (siRNA), microRNA (miRNA)) and NPs is a well-established technique known as PEGylation.^186^ We must note the importance of PEGylation in selective organ targeting of NPs, provides new insights into the structure–property relationship of LNPs, and offers a novel, simple, and practical PEGylation technology to prepare the next generation of safe and effective nanovaccines against COVID-19. Danijela Zukancic *et al.*,^187^ discovered that LNPs stabilized by 3% Tween 20, a surfactant with a branched PEG chain linking to a short lipid tail, achieved highly specific transfection at the lymph node.

## Conclusion and future perspectives

In this article, we summarized recent data about COVID-19, etiology, pathogenesis, prevention routes, diagnosis, and the currently-available medications. After that, we opened the way of new nanomaterial-based approaches to increase the bioavailability and efficiency of COVID-19 drugs by their incorporation inside and/or onto the surface of 5 promising nanomaterial-based drug delivery systems. Their preparation routes and unique advantages were mentioned. Then, we discussed in detail, one potential nanoemulsion system containing Avigan drug. The proposed action mechanism of this unique nano-based drug was explained in the light of solid references. Many research studies and clinical trials are still under investigation for the development of effective and safe cure for COVID-19, some trials showed good promise. However, more investigation is required. Nanotechnology can foster the development of new nanodrugs and nanovaccines by acting as effective drug delivery systems.

## Author contributions

MAE, and GSE conceived the concept and idea of the present review, worked on the study design strategy and selected the topics to be discussed, draws the figures, did literature searches and screened titles and abstracts for relevance, abstracted the data from the eligible full text articles, analyzed and interpreted the data, and drafted the manuscript, revised the final draft of the manuscript. SOO, and MM worked on the study design strategy and selected the topics to be discussed, and did literature searches and screened titles and abstracts for relevance. All authors have read and approved the final draft.

## Compliance with ethical standards

### Funding

Not applicable.

### Research involving human participation and/or animals

Not applicable.

### Informed consent

Not applicable.

### Ethical approval

Not applicable.

### Availability of data and materials

Not applicable.

## Conflicts of interest

The authors declare that they have no conflict of interest.

## Supplementary Material
